# Disparities, variations, inequalities or inequities: whatever you call them, we need data to monitor them

**DOI:** 10.1186/s13584-019-0307-7

**Published:** 2019-04-29

**Authors:** Jennifer S. Mindell

**Affiliations:** 0000000121901201 grid.83440.3b UCL, Gower Street, London, WC1E 6BT UK

**Keywords:** Disparities, Variations, Inequalities, Inequities, Sustainable development goals (SDGs)

## Abstract

Health inequalities are a problem in high, middle and low income countries. Most are unfair (‘inequities’) and could be minimised but primarily through policies outside the health service.

In the US, the Center for Diseases Control has used high quality, nationally-available data to monitor conditions and determinants of health among different groups (by sex, disability, race, ethnicity, and language) to motivate action to reduce inequalities. In the UK, the 10 top level ‘health’ indicators in London at the turn of the millennium included unemployment, education, housing quality, crime, air pollution, road travel injuries, as well as traditional health measures. Most of these affect mental and physical health through social determinants or adverse environmental exposures. Current inequalities monitoring in England includes a Local Basket of Inequalities Indicators focusing on a wide range of determinants of health as well as traditional health metrics.

Israel, like the US, has above average socio-economic inequalities but has universal healthcare. Health inequalities in Israel occur within different Jewish groups and by income, education, ethnicity, and religion, with disadvantages often clustering. Current monitoring in Israel focuses on health outcomes and ‘midstream’ healthcare-related provision. I agree with Abu-Saad and her colleagues that including monitoring of social determinants of health is crucial to identify and tackle health inequalities in Israel.

National, ‘upstream’, interventions are the most effective ways to reduce inequalities and improve the population’s health. High-level political support is crucial for this. While a ‘Health in all Policies’ approach combined with political will to ‘leave no one behind’ can lead to great improvements, regular monitoring is essential, to: identify the inequities; plan appropriate and effective, targeted interventions; implement and evaluate them; and change them where needed. All of this requires adequate and timely data on health and its determinants, including information about undiagnosed and poorly controlled disease, obtained from the general population not just those attending for healthcare, analysed for each population sub-group at risk of experiencing inequalities.

This is a commentary on 10.1186/s13584-018-0208-1

## Main text

### Disparities, inequalities, and inequities

On reading the paper by Abu-Saad, Avni and Kalter-Leibovici [1], my initial reaction to the title and abstract was one of dismay that the term ‘health disparities’ is being used in Israel, as it is in the USA [2]. The term reminds me of the Thatcher years in England, when ‘health variations’ was the term the government considered acceptable, disregarding considerations of social justice; those working for the government were banned from talking about ‘social inequalities’ [3, 4].

I believe that “inequality” is a more appropriate term than “disparity.” For example, during the Thatcher period in England, home ownership increased markedly. However, figures from the Institute for Fiscal Studies show that over the 11 years from 1979 to 1990, the mean income, adjusted for inflation, rose by 4.6% among the poorest decile of households but by 46.9% among the richest decile: the income in the latter group went from 3.1 times to 4.4 times that of the former, leading to a dramatic increase in socioeconomic inequalities during that time. The number of children living in poverty also increased from 1.7 million to 3.3 million [5]. All these inequalities have continued to increase since then [5].

According to the U.S. Centers for Disease Control and Prevention (CDC): *“Health equity is when everyone has the opportunity to be as healthy as possible”* and *“Health disparities are differences in health outcomes and their causes among groups of people”* [6]. In most countries worldwide, at least in public health circles, ‘inequalities’ refer to differences and ‘inequities’ to unfair differences, such as in a recent report from Canada [7].

Fortunately, once I reached the paper itself, my concern was allayed, as the main text opens with the statement that *“Health disparities or inequities are defined as ‘avoidable and unjust differences in exposure and vulnerability to health risk factors, health-care outcomes, and the social and economic consequences of these outcomes’”*, citing the World Health Organization [8]*.*

### Health inequities in Israel and the U.S.

The paper by Abu-Saad and colleagues focuses on the U.S., noting its lack of national healthcare insurance and its high poverty and income inequality relative to most OECD countries, and draws lessons on monitoring the effectiveness of attempts to reduce these inequities. Israel resembles the U.S. regarding the above average extent of inequalities and resembles the UK (which also has inequalities by age, gender, income, and ethnicity [9, 10]) in having universal healthcare. Abu-Saad et al. provide evidence of the health inequities in Israel within the different Jewish groups as well as between the broader majority and minority populations, mentioning the collinearity in many cases between income, education, ethnicity, and religion. Indeed, Daoud et al. showed that after adjusting for socio-economic differences in education and income, Arabs in Israel had better self-reported health than longstanding Jewish residents [11].

The paper then describes the impressive series of ten-yearly *Healthy People* goals in the U.S., with the changing priorities each decade. However, having so many priorities can result in no priorities in practice. Therefore, ‘Leading health indicators’ were identified by CDC, using data that were high quality; available nationally; monitored conditions or determinants of health that were of public health importance or where effective, feasible interventions exist for a health condition suffered inequitably by different groups; and where the indicators could motivate action.

While Israeli efforts to reduce health inequities began about a decade ago, those in the U.S. started in the 1980s. However, it was not until 2011 that the U.S. authorities set consistent definitions and minimum data standards for the long list of parameters associated with health inequalities, including sex, disability, race, ethnicity, and language.

### ‘Upstream’ and ‘downstream’ policies

Hosseinpoor et al. noted that while the Millennium Development Goals focused on reduced inequalities between countries, the Sustainable Development Goals (SDGs) aim to reduce inequalities within countries [12]. It is ironic that both the Millennium and Sustainable Development Goals were intended primarily for low and middle income countries, yet some of the worst inequities occur in the U.S. [13]. The problem is, of course, that most health inequities are related primarily to social determinants of health [14], requiring political will to address these.

While Abu-Saad and colleagues report on high-level political support in the U.S. from the mid-1980s and subsequently [1], ongoing support seems unlikely in the current political climates in the U.S., Israel, and the UK, except within the limits of continuing legal requirements. This is particularly pertinent given the findings by many non-governmental organisations, reported by Abu-Saad et al., that early initiatives tended to focus on individual behaviours and healthcare [1], both known to be the least effective ways of reducing inequalities compared with national, ‘upstream’, interventions.

For example, Gillespie et al. compared two approaches to reducing salt intake in the population in the UK, where about 80–85% of sodium intake comes from manufactured food. They estimated that mandatory reformulation of manufactured food would have ten times more impact than social marketing to influence individual behaviour would. More importantly, in the context of inequalities, mandatory reformulation would have a 49% greater effect in the most deprived compared with the most affluent [15] whereas social marketing would not reduce health inequalities (and could actually increase them).

### Social determinants of health

In most countries, there are no inequalities in polio because polio has been eradicated. That is also the desired endpoint for smoking and other health-harming behaviours; universal adoption of activities benefitting health, including preventive care, early diagnosis, and effective management would eradicate healthcare-driven inequalities. However, as mentioned above, most health inequalities are driven by inequalities in the determinants of health.

The ‘health’ indicators used in London almost two decades ago took a different approach to monitoring health and inequalities in London than either the US approach or what Abu-Saad et al. suggest. The 10 top level ‘health’ indicators in London were: unemployment overall and among black and minority ethnic people; educational attainment; the proportion of homes judged unfit to live in; the domestic burglary rate; air quality indicators (NO_2_ and PM_10_); road traffic injury rate; life expectancy at birth; infant mortality rate; and the proportion of people with self-assessed good health [16]. Most of these are factors that affect mental and physical health through social determinants (education, unemployment, housing, security) or adverse environmental exposures (air pollution) rather than direct measures of poor – or good – health.

In the past decade there have been changes in approach by *Healthy People 2020* and CDC to include monitoring inequalities in social determinants of health. Abu-Saad et al. report the critique of such indicators, which compartmentalise socio-economic inequity rather than considering the clustering and co-existence of many aspects of disadvantage within certain groups. They recommend that Israel should include social determinants of health as outcomes/indicators of inequality in addition to outcome measures more immediately recognisable as ‘health’ indicators [1]. I would encourage an approach that follows the example of London from the turn of the millennium, as it can focus political minds on health and its social determinants, not just on healthcare.

Current monitoring of national, regional and local level inequalities in England include the Compendium of Indicators produced by NHS Digital [17] and Public Health England (PHE)‘s Outcomes Frameworks [18]. The former includes data on cancer, public health, area deprivation, and the Local Basket of Inequalities Indicators, which includes unemployment, poverty, housing, homelessness, education, crime, pollution, community development, lifestyle behavioural risk factors for chronic non-communicable diseases (NCDs), access to healthcare, injuries, mental health, maternal and child health, older people, and tackling NCDs [19]. PHE’s Outcomes Framework, published quarterly, aims to support public health efforts to improve the public’s health, *“and to improve the health of the poorest fastest”* [18]. However, the problem remains that the determinants of inequalities lie primarily within the remit of national government, although local government policies can improve or exacerbate such inequalities.

### Health examination surveys

One aspect missing from Abu-Saad, Avni and Kalter-Leibovici’s account of monitoring health inequalities and their list of indicators used in the U.S. is the consideration of undiagnosed disease. This cannot be detected using healthcare data, nor from health interview surveys, but requires biophysical measurements of a random sample of the general population [20]. Such surveys are now routine in many countries across Europe [21] and other high and middle income countries (e.g. across Latin America [22]) and also in low income countries (e.g. in sub-Saharan Africa [23, 24]). A health examination survey (HES) costs more to run than a health interview survey but the information is more valuable, with documented examples of use in national policy-making [25, 26]. Inequalities exist in the prevalence of NCDs [9, 27, 28], related to inequalities in risk factors and in socio-economic and other circumstances [29], and in obtaining a diagnosis in the presence of disease [27, 30], related at least in part to the availability of healthcare insurance [30], but demographic and socio-economic inequalities in late diagnosis occur even in high income countries with universal healthcare, such as Israel and the UK [31].

Like many countries, Israel had a health interview survey (in 2004) and more recently as part of the European Health Interview survey (EHIS) but had no health examination survey undertaken. This has changed, to a limited extent, by the development of MABAT, the National Health and Nutrition survey, based on a random sample of the general population. As with the Health Survey for England, different surveys have had a different focus. MABAT zahav, 2005–06, was limited to people aged 65+ who were members of either of the two largest HMOs in Israel, covering 87% of the Israeli population of that age. Handgrip was also measured in these participants, as it was in people aged 65+ participating in the HSE 2005.

MABAT includes anthropometric measurements to obtain an accurate assessment of obesity prevalence [32]. Blood pressure was also measured so the prevalence of undiagnosed hypertension can be estimated, using measurements in conjunction with information from asking about (self-reported) doctor-diagnosed high blood pressure. This is thus comparable with the approaches taken in most health examination surveys but a more limited range of undiagnosed diseases can be detected in MABAT compared with the UK and USA. Information on undiagnosed disease (including, for example, a measure of blood glucose or serum creatinine for diabetes or kidney disease) would also help to target interventions to reduce health inequalities due to unequal use of healthcare, as there is more to access and equitable use of healthcare than it being freely available [33], even after adjustment for the increased need in poorer people [34, 35].

## Conclusions

Health inequalities are a problem in high, middle and low income countries. Most are unfair (‘inequities’) and could be minimised but primarily through policies outside the health service. While a ‘Health in all Policies’ approach combined with political will to ‘leave no one behind’ can lead to great improvements, regular monitoring is essential to: identify the inequities; plan appropriate and effective interventions; implement and target them; evaluate them; and change them where needed. All of this requires adequate and timely data, obtained from the general population using health examination surveys in addition to healthcare data, to ensure undiagnosed disease in included.

## Abbreviations

CDC: U.S. Centers for Disease Control and Prevention
NCDs: Chronic non-communicable diseases
NHS: National Health Service
PHE: Public Health England
SDGs: Sustainable Development Goals
